# The quality of drinking and domestic water from the surface water sources (lakes, rivers, irrigation canals and ponds) and springs in cholera prone communities of Uganda: an analysis of vital physicochemical parameters

**DOI:** 10.1186/s12889-020-09186-3

**Published:** 2020-07-17

**Authors:** Godfrey Bwire, David A. Sack, Atek Kagirita, Tonny Obala, Amanda K. Debes, Malathi Ram, Henry Komakech, Christine Marie George, Christopher Garimoi Orach

**Affiliations:** 1 Department of Community and Behavioral Sciences, Makerere University College of Health Sciences, School of Public Health, Kampala, Uganda; 2 Department of International Health, Johns Hopkins Bloomberg School of Public Health, Dove Project, Baltimore, MD USA; 3 Uganda National Health Laboratory Services (UNHS/CPHL), Ministry of Health, Kampala, Uganda; 4 Department of Quality Control, Uganda National Drug Authority, Kampala, Uganda

**Keywords:** Drinking water, Cholera, Africa, Uganda, Water quality, Physicochemical parameter, Water source, Surface water, Lake, Safe water

## Abstract

**Background:**

Water is the most abundant resource on earth, however water scarcity affects more than 40% of people worldwide. Access to safe drinking water is a basic human right and is a United Nations Sustainable Development Goal (SDG) 6. Globally, waterborne diseases such as cholera are responsible for over two million deaths annually. Cholera is a major cause of ill-health in Africa and Uganda. This study aimed to determine the physicochemical characteristics of the surface and spring water in cholera endemic communities of Uganda in order to promote access to safe drinking water.

**Methods:**

A longitudinal study was carried out between February 2015 and January 2016 in cholera prone communities of Uganda. Surface and spring water used for domestic purposes including drinking from 27 sites (lakes, rivers, irrigation canal, springs and ponds) were tested monthly to determine the vital physicochemical parameters, namely pH, temperature, dissolved oxygen, conductivity and turbidity.

**Results:**

Overall, 318 water samples were tested. Twenty-six percent (36/135) of the tested samples had mean test results that were outside the World Health Organization (WHO) recommended drinking water range. All sites (100%, 27/27) had mean water turbidity values greater than the WHO drinking water recommended standards and the temperature of above 17 °C. In addition, 27% (3/11) of the lake sites and 2/5 of the ponds had pH and dissolved oxygen respectively outside the WHO recommended range of 6.5–8.5 for pH and less than 5 mg/L for dissolved oxygen. These physicochemical conditions were ideal for survival of *Vibrio. cholerae*.

**Conclusions:**

This study showed that surface water and springs in the study area were unsafe for drinking and had favourable physicochemical parameters for propagation of waterborne diseases including cholera. Therefore, for Uganda to attain the SDG 6 targets and to eliminate cholera by 2030, more efforts are needed to promote access to safe drinking water. Also, since this study only established the vital water physicochemical parameters, further studies are recommended to determine the other water physicochemical parameters such as the nitrates and copper. Studies are also needed to establish the causal-effect relationship between *V. cholerae* and the physicochemical parameters.

## Background

Water is the most abundant resource on the planet earth [1], however its scarcity affects more than 40% of the people around the world [2]. Natural water is an important material for the life of both animals and plants on the earth [3]. Consequently, access to safe drinking water is essential for health and a basic human right that is integral to the United Nations Resolution 64/292 of 2010 [4]. The United Nations set 2030 as the timeline for all countries and people to have universal access to safe drinking water; this is a Sustainable Development Goal (SDG) 6 of the 17 SDGs [5]. The availability of and access to safe water is more important to existence in Africa than it is elsewhere in the world [6]. Least Developed Countries (LDCs) especially in sub-Saharan Africa have the lowest access to safe drinking water [7]. In Africa, rural residents have far less access to safe drinking water and sanitation than their urban counterparts [8].

Natural water exists in three forms namely; ground water, rain water and surface water. Of the three forms, surface water is the most accessible. Worldwide, 144 million people depend on surface water for their survival [9]. In Uganda, 7% of the population depends on surface water (lakes, rivers, irrigation canal, ponds) for drinking water [10]. The same surface water is a natural habitat for many living organisms [11] some of which are responsible for transmission of infectious diseases such as cholera, typhoid, dysentery, guinea worm among others [12]. Surface water sources include lakes, rivers, streams, canals, and ponds. These surface water sources are often vulnerable to contamination by human, animal activities and weather (storms or heavy rain) [13, 14]. Globally, waterborne diseases such as diarrheal are responsible for more than two million deaths annually. The majority of these deaths occur among children under-5 years of age [15].

Cholera, a waterborne disease causes many deaths each year in Africa, Asia and Latin America [16]. In 2018 alone, a total of 120,652 cholera cases and 2436 deaths were reported from 17 African countries to World Health Organization [17]. Cholera is a major cause of morbidity and mortality in Uganda [18]. The fishing communities located along the major lakes and the rivers in the African Great Lakes basin of Uganda constitutes 5% of the Uganda’s population, however these communities were responsible for the majority (58%) of the reported cholera cases during the period 2011–2015 [19]. Cholera outbreaks affect predominantly communities using the surface water and the springs. There is also high risk of waterborne disease outbreaks in the communities using these types of water [20, 21]. Studies of the surface water from water sources located in the lake basins of the five African Great Lakes in Uganda identified *Vibrio. cholerae* [22, 23] though no study isolated the toxigenic *V. cholerae* O1 or O139 that cause epidemic cholera. Cholera outbreaks in the African Great Lakes basins in Uganda have been shown to be propagated through water contaminated with sewage [20, 24]. Cholera is one of the diseases targeted for elimination globally by the WHO by 2030 [25]. Hence, to prevent and control cholera outbreaks in these communities, promotion of use of safe water (both quantity and quality), improved sanitation and hygiene are the interventions prioritized by the Uganda Ministry of Health [26]. Most importantly, provision of adequate safe water is a major pillar of an effective cholera prevention program given that water is the main mode of *V. cholerae* transmission [27, 28].

Availability of adequate safe water is essential for prevention of enteric diseases including cholera [29]. Therefore, access to safe drinking and domestic water in terms of quantity and quality is key to cholera prevention. Water quality is defined in terms of three key quality parameters namely, physical, chemical and microbiological characteristics [30]. A less common but important parameter is the radiological characteristics [31]. In regards to the physicochemical parameters, there are five parameters that are essential and impacts life (both flora and or fauna) within the aquatic systems [32]. These vital physicochemical parameters include pH, temperature, dissolved oxygen, conductivity and turbidity [32].

pH is a value that is based on logarithm scale of 0–14 [33]. Aquatic organisms prefer pH range of 6.5–8.5 [34–36]. Low pH can cause the release of toxic elements or compounds into the water [37]. The optimal pH for *V. cholerae* survival is in basic range (above 7). *Vibrio cholerae* may not survive for long in acidic pH [38]. A solution of pH below 4.5 will kill *V.cholerae* bacteria [39].

Most aquatic organisms are adapted to live in a narrow temperature range and they die when the temperature is too low or too high [34]. *Vibrio cholerae*, bacteria proliferate during algae bloom resulting in cholera outbreaks [40, 41]. This proliferation could be due favourable warm temperature [42]. Relatedly, *V. cholerae* isolation from natural water in endemic settings is strongly correlated with water temperature above 17 °C [43].

Dissolved oxygen is the oxygen present in water that is available to aquatic organisms [34]. Dissolved oxygen is measured in parts per million (ppm) or milligrams per litre (mg/L) [35]. Organisms in water need oxygen in order to survive [44]. Decomposition of organic materials and sewage are major causes of low dissolved oxygen in water [12].

Water conductivity is the ability of water to pass an electrical current and is expressed as millisiemens per metre (1 mS m-^1^ = 10 μS cm^− 1^) [29]. Most aquatic organisms can only tolerate a specific conductivity range [45]. Water conductivity increases with raising temperature [46]. There is no set standard for water conductivity [45]. Freshwater sources have conductivity of 100 – 2000μS cm^− 1^. High water conductivity may be due to inorganic dissolved solids [46].

Turbidity is an optical determination of water clarity [47]. Turbidity can come from suspended sediment such as silt or clay [48]. High levels of total suspended solids will increase water temperatures and decrease dissolved oxygen (DO) levels [12]. In addition, some pathogens like *V. cholerae, Giardia lambdia* and *Cryptosporidia* exploit the high water turbidity to hide from the effect of water treatment agents and cause waterborne diseases [49]. Consequently, high water turbidity can promotes the development of harmful algal blooms [41, 50].

Given the importance of the water physicochemical parameters, in order to ensure that they are within the acceptable limits, the WHO recommends that they are monitored regularly [51]. The recommended physicochemical parameters range for raw water are for pH of 6.5–8.5, turbidity of less than 5Nephlometric Units (NTU) and dissolved oxygen of not less than 5 mg/L [51]. Surface and spring water with turbidity that exceeds 5NTU should be treated to remove suspended matter before disinfection by either sedimentation (coagulation and flocculation) and or filtration [52].

Water chlorination using chlorine tablets or other chlorine releasing reagent is one of the most common methods employed to disinfect drinking water [53, 54]. Chlorination is an important component of cholera prevention and control program [55]. In addition to disinfection to kill the pathogens, drinking water should also be safe in terms of physicochemical parameters as recommended by WHO [51]. However, to effectively make the water safe using chlorine tablets and other reagents, knowledge of the physicochemical properties of the surface and spring water being disinfected is important as several of the parameters affect the active component in the chlorine tablets [56]. For example, chlorine is not effective for water with pH above 8.5 or turbidity of above 5NTU [53].

Generally, there is scarcity of information about the quality and safety of drinking water in Africa [57]. Similarly, few studies exist on the physicochemical characteristics of the drinking water and water in general in Uganda. Furthermore, information from such studies is inadequate for use to increase safe water in cholera prone districts of Uganda where the need is greatest. The cholera endemic communities of Uganda [19, 21, 24] have adequate quantities of water that is often collected from the Great lakes, rivers and other surface water sources located within the lake basins. However, the water is of poor quality in terms of physicochemical and microbiological characteristics. Several studies conducted in Uganda have documented microbiological contamination of drinking water [20, 24, 58, 59]. However, few studies exist on the physicochemical characteristics of these water. Furthermore, these studies focused on few water sources, for example testing the lakes and omitted the rivers, springs and ponds or testing the rivers and omitted the other water types. One such study was carried out on the water from the three lakes in western Rift valley and Lake Victoria in Uganda [23], This study did not assess the other common water sources such as the rivers, ponds and springs that were used by the communities for drinking and other household purposes. Other studies on water physicochemical characteristics assessed heavy metal water pollution of River Rwizi (Mbarara district, Western Uganda) [60] and of the drinking water (bottled, ground and tap water) in Kampala City (Central Uganda) [61] and Bushenyi district (Western Uganda) [62]. These studies found high heavy metal water pollution in the drinking water tested. The information gathered from such studies is useful in specific study area and is inadequate to address the lack of safe water in the cholera endemic districts of Uganda where the need for safe drinking water is greatest. Several epidemiological studies in Uganda have attributed cholera outbreaks to use of contaminated surface water [20, 21, 24, 63]. Furthermore, studies conducted on the surface water focus on pathogen identification [63, 64] leaving out the water physicochemical parameters which are equally important in the provision of safe drinking water [53] and are necessary for survival of all living organisms (both animals and plants) [44].

Therefore, the aim of this study was to determine the physicochemical characteristics of the surface water sources and springs located in African Great Lakes basins in Uganda so as to guide the interventions for provision of safe water to cholera prone populations [19–21, 24, 58] of Uganda. This study in addition has the potential to guide Uganda to attain the United Nations SDG 6 target of universal access to safe drinking water [2] and the WHO cholera elimination Roadmap [25] by 2030. Furthermore, these findings may guide future studies including those on causal-effect relationship between physicochemical parameters and infectious agents (pathogens).

## Methods

**Fig. 1:**
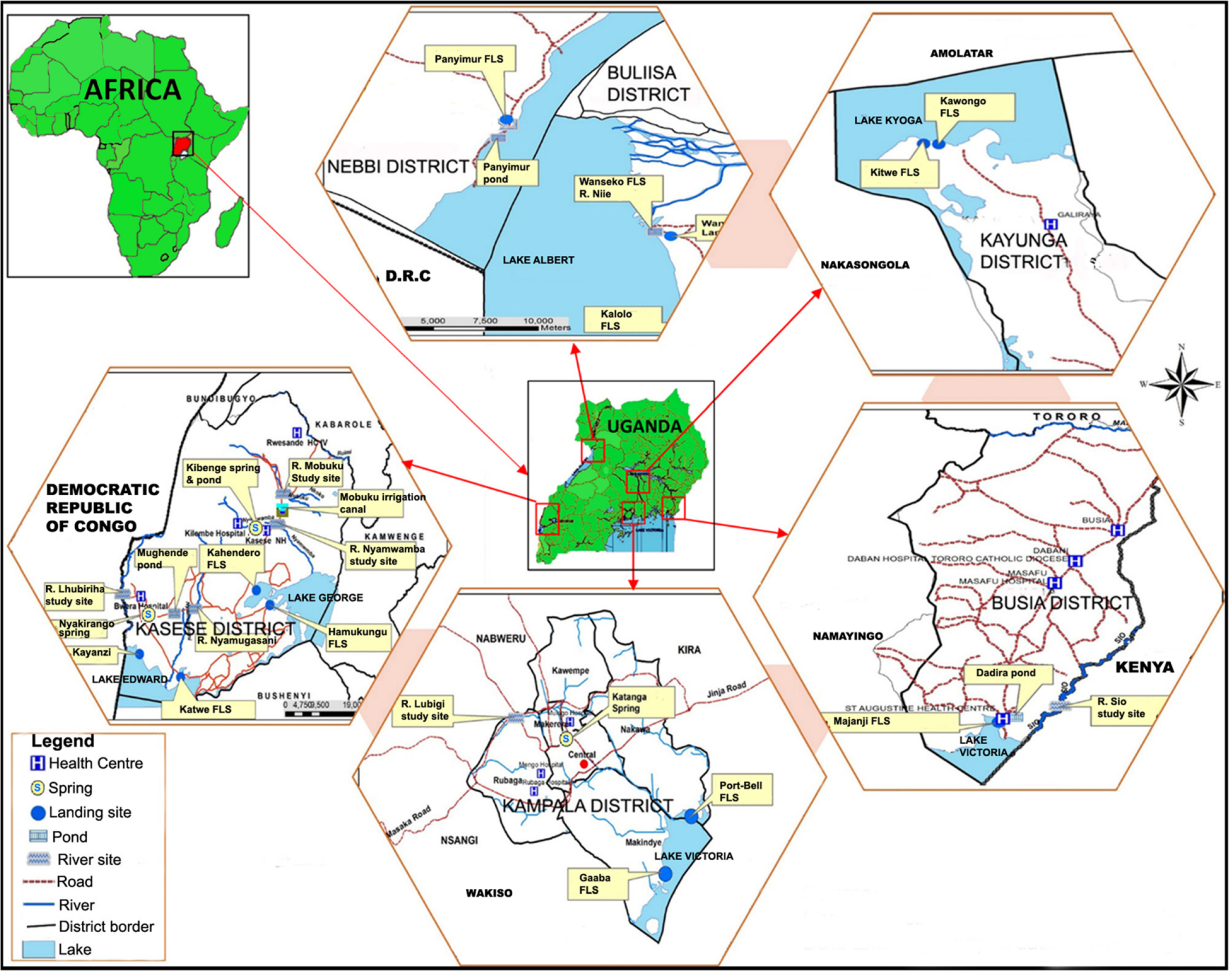
Map showing the location of Uganda, the study districts, major surface water sources and the study sites, February 2015 – January 2016. The blue shades are the African Great Lakes and their basins. (Map generated by ArcGIS version 10.2 [licenced] and assembled using Microsoft Office PowerPoint, Version 2016 [licenced] by the authors)

This was a longitudinal study that was conducted between February 2015 and January 2016 in six districts of Uganda that are located in the African Great Lakes basins of the five lakes (Victoria, Albert, Kyoga, Edward and George). These districts had ongoing cholera outbreaks or history of cholera outbreaks in the previous five to 10 years (2005–2015). In addition, the selected study districts had border access to the following major water bodies (lakes: Victoria, Albert, Edward, George and Kyoga). The study area was purposively selected because the communities residing along these major lakes contributed most (58%) of the reported cholera cases and deaths in Uganda [19, 65] and in the sub-Saharan Africa region [66] in the past 10 years. Water samples were collected monthly from 27 sites used by the communities for household purposes that included drinking. Water samples were then tested to determine the vital physicochemical parameters. The water samples were collected from lakes, rivers, springs, ponds and an irrigation canal that were located in the lake basins of the five African Great Lakes in Uganda. In one site, water was also collected from a nearby drainage channel and tested *for V. cholerae* [22] and physicochemical parameters. However, because the channel was not used for drinking the results were omitted in this article. Water samples were analysed to determine the pH, temperature, dissolve oxygen, conductivity and turbidity. The study sites were located in the districts of Kampala and Kayunga in central region of Uganda; Kasese and Buliisa districts in western Uganda; Nebbi and Busia districts in northern and eastern Uganda respectively. The study sites were the same as for the simultaneous bacteriological *V. cholerae* detection study [22] and are shown in Fig. 1**.**

### Rural-urban categorization of the study sites

The study sites were categorized as urban if they were found in Kampala district (the Capital City of Uganda) or rural if they were in the other five remote study districts (Kasese, Kayunga, Busia, Nebbi and Buliisa).

### Identification of the study sites and water testing procedures

The sites for water testing were identified with the guidance of the local communities and after direct observation by the study team. Geo-coordinates of the sites were taken at the beginning of the study to ensure that subsequent water collection and measurements were done on water from specific points. Two water collection sites were selected on each of the African Great Lakes in Uganda. The selected sites were in different locations but within the communities with a history of cholera outbreaks in the previous 10 years prior to the study period. For each selected lake point, a site was also selected on a river, a spring and a pond located within the area and being used by the communities for domestic purposes that included drinking and preparation of food. A total of 27 sites, two of which were from each of the five lakes were selected and the water tested. The number of sites on each lake and their locations are shown in Additional file 1.

Water samples were collected and tested monthly for 12 months by the research assistants who were health workers with background training in microbiology or environmental health. The research assistants received training on water collection and testing from a water engineer. The physicochemical parameters were measured by use of the digital meters namely the *Hach meter HQ40d* and digital turbidity meter.

Water samples were collected in five-litre containers, three litres were processed for *V. cholerae* detection by Polymerase Chain Reaction (PCR) test as previously described [67]. *Vibrio cholerae* Non O1/Non O139 pathogens were frequently detected in the water samples during the study period [22]. While the three litres of water were being processed *for V. cholerae* detection [22], the rest of the water (2 l), were simultaneously used for the onsite measurement of temperature, pH, conductivity and dissolved oxygen. The *Hach* meters*,* HQ40d used in the study, had three electrodes that were calibrated before each monthly testing according to the manufacturers’ manual [68]. The *Hach* meter calibrations were done using three specific standard buffer solutions that were for pH, dissolved oxygen and conductivity respectively. Turbidity (total suspended solids or water clarity) was measured using a turbidity meter according to previously published methods [49]. In addition, the research assistants were provided with Standard Operating Procedures (SOPs) and supervised monthly by the investigators before and during each scheduled monthly measurements.

### Data management, analysis and statistical tests

Data were collected, entered, cleaned and stored in the spreadsheet. Errors in the recorded readings were removed using the correct records retrieved from the *Hach meters’ HQ40d* internal memory. Stata statistical package version 13 was used to analyse the data. Data were analysed to generate means and standard error of the mean for pH, temperature, dissolved oxygen (DO), conductivity (CD) and turbidity. Data were presented in the form of tables and graphs. Comparison for variations between the water samples were carried out using One-Way Analysis of Variance (ANOVA) test. Samples with significant One-Way ANOVA test were subjected to Turkey’s Post Hoc test to establish which of the variables were statistically significant.

The map was created using ArcGIS software, Version 10.2, licenced (ESRI, Redlands, California, USA). The graphs and figures were produced using Microsoft Excel and PowerPoints, Version 2016 (Microsoft, Redmond, Washington, USA). The administrative shapefiles used to create the map were obtained from open access domain, the Humanitarian Data Exchange: https://data.humdata.org/. In order to generate the study locations on the map, Global Positioning System (GPS) coordinates for the study sites were converted to shapefiles that were combined with the administrative shapefiles corresponding to the locations.

## Results

A total of 318 water samples were tested from 27 sites as follows; lake water 40.9%, (130/318), rivers water 26.4% (84/318), ponds water 17.9% (57/318), spring water 11.0% (35/318) and canal water 3.8% (12/318).

### Test results for the lake water collected at the fish landing sites (FLS)

The mean physicochemical test results for pH, temperature, dissolved oxygen, conductivity and turbidity are shown in Table 1**.**

**Table 1:** The mean physicochemical characteristics of lake water in the study area, February 2015 – January 2016

Name of the fish landing site (FLS)	pH	Temp (°C)	DO (mg/L)	CD (μS/cm)	Turb (TNU)
**WHO Acceptable levels**	**6.5–8.5**	**–**	**> 5**	**–**	**0–5**
*Kalolo (L. Albert)*	7.18 ± 0.29	28.09 ± 0.86	5.05 ± 0.70	668.18 ± 186.76	167.29 ± 22.51
*Panyimur L. Albert)*	7.46 ± 0.33	29.65 ± 0.52	5.90 ± 0.34	748.83 ± 182.73	94.17 ± 28.95
*Katwe salt (L. Edward)*	8.57 ± 0.14	31.69 ± 0.44	6.79 ± 0.26	574.25 ± 26.19	105.53 ± 36.25
*Kayanzi (L. Edward)*	7.08 ± 0.06	24.19 ± 0.79	6.76 ± 0.22	80.33 ± 3.33	67.39 ± 10.78
*Hamukungu (L. George)*	9.03 ± 0.17	28.83 ± 1.02	6.81 ± 0.21	274.92 ± 9.42	124.29 ± 27.02
*Kahendero (L. George)*	9.13 ± 0.23	30.79 ± 1.01	6.56 ± 0.20	333.65 ± 9.91	175.39 ± 33.75
*Kawongo (L. Kyoga)*	7.27 ± 0.23	28.07 ± 0.35	6.54 ± 0.16	124.87 ± 12.42	136.03 ± 32.51
*Kitwe (L. Kyoga)*	7.10 ± 0.16	28.44 ± 0.46	5.90 ± 0.50	91.90 ± 8.96	88.13 ± 17.96
*Majanji (L. victoria)*	7.41 ± 0.13	28.24 ± 0.54	6.76 ± 0.32	111.05 ± 31.05	6.37 ± 2.55
*Gaaba (L. Victoria)*	6.80 ± 0.42	27.80 ± 0.53	6.04 ± 0.62	193.23 ± 45.93	67.11 ± 17.74
*Port Bell (L. Victoria)*	6.60 ± 0.23	27.17 ± 0.84	4.78 ± 0.45	190.29 ± 49.35	63.09 ± 14.70

***NB: values in the table are mean ± standard error***

***Temp*Temperature,*DO*Dissolved Oxygen,*Turb*Turbidity,*pH*pH,*CD*Conductivity,*FLS*Fish Landing Site**

The mean physicochemical water characteristics of most of the sites were within the WHO recommended water safety range except for turbidity. Few sites had pH and dissolved oxygen outside the WHO recommended safety range.

### Monthly variations of the lake water physicochemical characteristics

There were monthly variations in the physicochemical parameters between the water from the lake sites overtime. Most of the sites had steady pH overtime for the first half of the study period (February – August 2015). Thereafter, the pH reduced slightly during the second half (September, 2015 – January, 2016) of the study period. The highest pH fluctuations were in the months of October – December, 2015. The widest change in pH within the same site was observed at Gaaba Fish landing site, Lake Victoria basin, Kampala district.

There were differences in water temperature on the same lake but at different test sites. These differences were detectable mostly in the months of April, 2015. The lowest and highest water temperatures were both recorded on Lake Edward (Kasese district) at Kayanzi fish landing site of 18.9 °C and at Katwe FLS of 34 **°**C in the period between April – August, 2015. Fluctuations in the dissolved oxygen were detectable throughout the study period. Kalolo Fish landing site on Lake Albert, Buliisa district showed the widest fluctuations in dissolved oxygen with the highest value of 10.73 mg/L and the lowest of 2.5 mg/L.

**Fig. 2:**
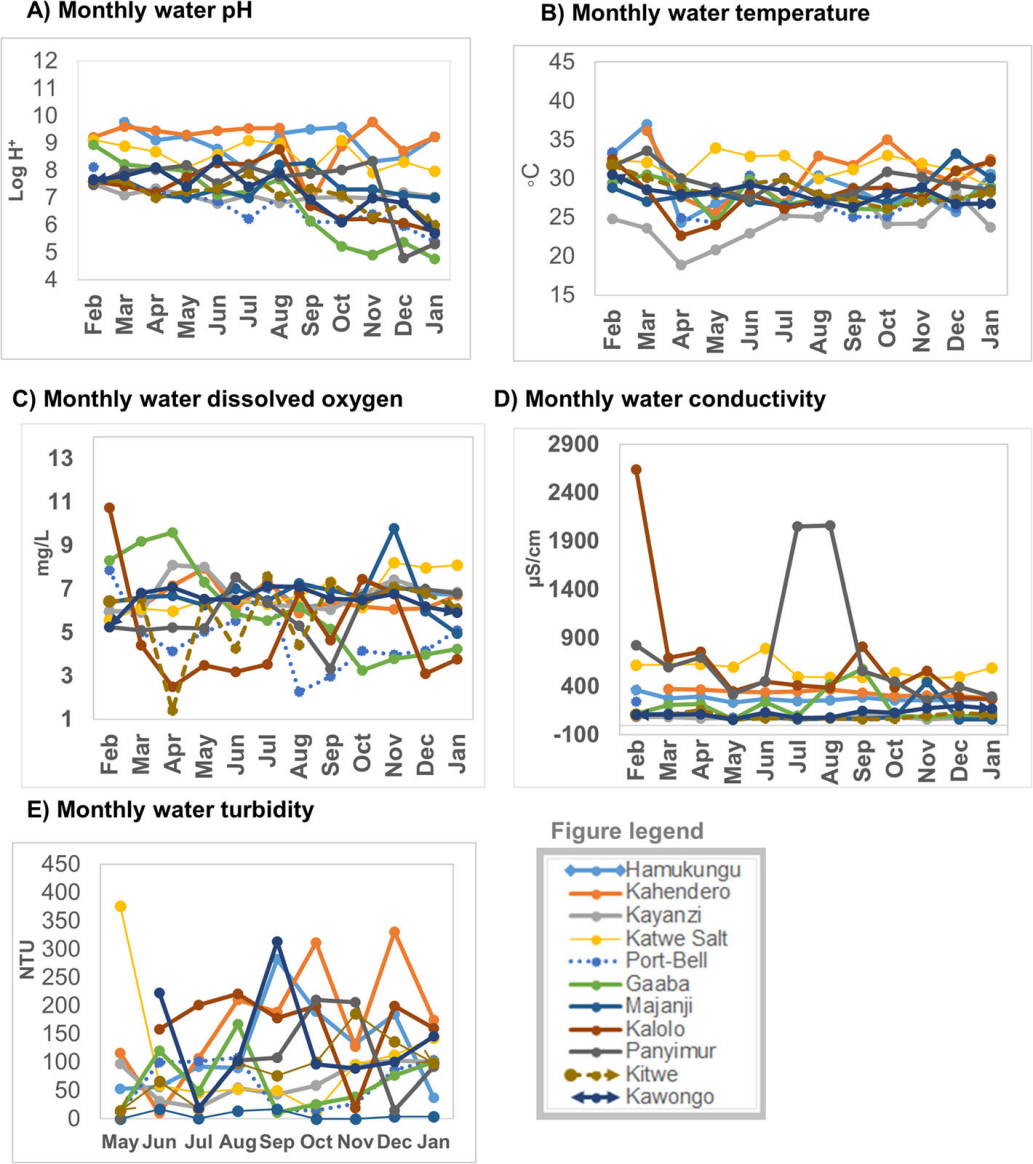
Monthly variations of lake water physicochemical characteristics (pH, temperature, dissolved oxygen, conductivity and turbidity), February 2015 – January 2016: Part **a**) water pH variations; Part **b**) water temperature variations; Part **c**) water dissolved oxygen; Part **d**) water conductivity variations; Part **e**) water turbidity variations

Most test sites had small conductivity fluctuations except for Panyimur and Kalolo both of which were located on Lake Albert in Nebbi and Buliisa districts These districts had high water conductivity fluctuations with arrange of 267.1 μS/cm – 2640 μS/cm at Kalolo (Buliisa district) FLS and 296 μS/cm – 2061 μS/cm at Panyimur (Nebbi district). Water turbidity for the majority of the sites changed overtime. Kahendero fish landing site (Lake George, Kasese district) had the highest turbidity which was most noticeable in the months of October 2015 to January 2016. Majanji fish landing site (Lake Victoria, Busia district) had the lowest and most stable water turbidity. Monthly variations of the lake water physicochemical parameters are shown in Fig. 2**.**

### River water physicochemical parameter test results

The mean physicochemical characteristics of water from the seven rivers studied are shown in Table 2**.**

**Table 2:** Mean physicochemical characteristics of the water samples from the rivers in the study area, February 2015 – January 2016

River	pH	Temp (°C)	DO (mg/L)	CD (μS/cm)	Turb (TNU)
WHO Acceptable levels	**6.5–8.5**	**–**	**> 5**	**–**	**0–5**
*R. Lhubiriha*	7.07 ± 0.12	26.22 ± 1.38	6.91 ± 0.18	80.44 ± 7.49	72.30 ± 15.46
*R. Lubigi*	6.92 ± 0.17	24.53 ± 0.78	2.50 ± 0.65	460.51 ± 57.83	122.09 ± 23.78
*R. Mobuku*	7.32 ± 0.05	22.08 ± 0.80	6.52 ± 0.16	63.15 ± 5.07	7.93 ± 6.92
*R. Nyamugasani*	6.86 ± 0.07	27.83 ± 0.73	6.63 ± 0.19	946.08 ± 3.63	11.37 ± 0.96
*R. Nyamwamba*	7.28 ± 0.13	27.92 ± 1.34	6.22 ± 0.14	99.91 ± 8.13	24.06 ± 7.38
*R. Sio*	7.11 ± 0.09	26.90 ± 0.31	5.53 ± 0.22	118.57 ± 32.13	136.53 ± 36.44
*R. Victoria Nile*	6.94 ± 0.10	28.21 ± 0.66	5.21 ± 0.46	140.82 ± 22.01	54.62 ± 28.46

*NB: values in the table are mean ± standard error*

*Temp* Temperature, *DO* Dissolved Oxygen, *Turb* Turbidity, *pH* pH, *CD* Conductivity, *FLS* = Fish Landing Site

There were variations in the mean pH, temperature, dissolved oxygen and conductivity between study sites on the rivers. However, these mean parameter variations were in WHO acceptable drinking water safety limit except for River Lubigi, Kampala district which had mean dissolved oxygen below the recommended WHO range. At one time (February, 2015) River Lubigi had dissolved oxygen of 0.45 mg/L. The river water turbidity for all the test sites were above that recommended by WHO of less than 5NTU.

### Monthly variations of the river water physicochemical characteristics

**Fig. 3:**
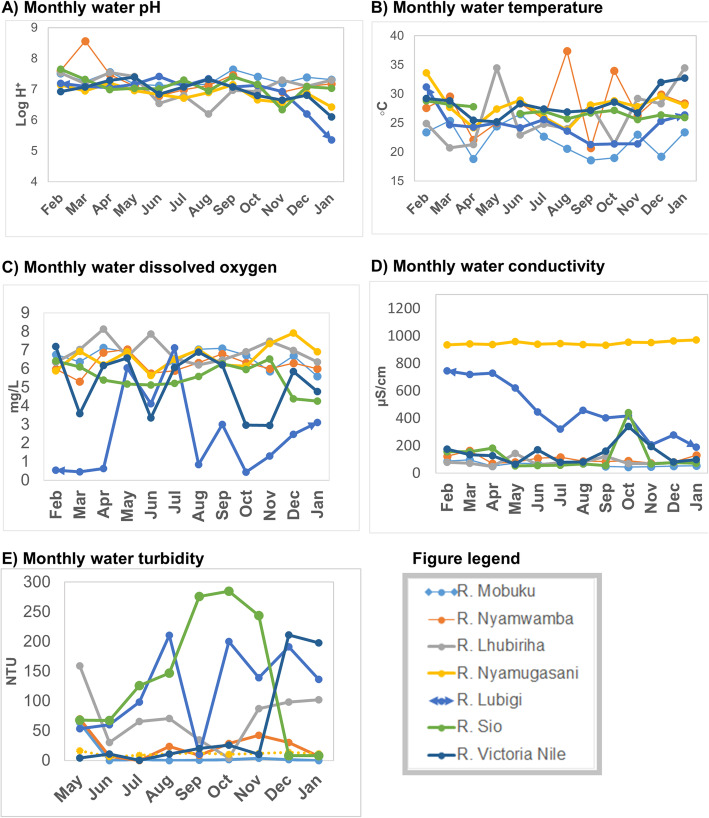
Monthly variations of the physicochemical characteristics of river water, February 2015 – January 2016: Part **a**) water pH variations; Part **b**) water temperature variations; Part **c**) water dissolved oxygen variations; Part **d**) water conductivity variations; Part **e**) water turbidity variations

Monthly variations in the water physicochemical characteristics of the seven river test sites are shown in Fig. 3**.**

There were variations in the water physicochemical parameters between rivers and within the same river overtime. Most rivers showed fluctuations of water pH and temperature. Some rivers such as R. Nyamugasani and R. Lhubiriha both in Kasese district had wide temperature fluctuations. River Mobuku (Kasese district) had the lowest water temperature recorded over the study period. Fluctuations in dissolved oxygen were highest in R. Lubigi (Kampala district), Lake Victoria basin. Dissolved oxygen for R. Lubigi was below the recommended level of more than 5 mg/L for most of the study period. Seasonal variations of water dissolved oxygen were also more noticeable in R. Lubigi than the rest of the river sites. Relatively more dissolved oxygen was found during the rainy seasons (March – July, 2015, first rainy season and September – December, 2015, second rainy season) than in dry season.

There were small variations in the water conductivity in the majority of the rivers. Wide fluctuations in conductivity were observed for water samples of R, Lubigi (Kampala district). River Nyamugasani (Kasese district, Lake Edward basin) had steady but higher conductivity than all the other rivers. There were variations in turbidity within the same river overtime and between the different rivers. River Sio (Busia district) had the highest and the widest turbidity variations during the study period.

### Water test results for the springs and ponds

The mean physicochemical characteristics of spring and pond water are shown in Table 3**.**

**Table 3:** The mean physicochemical characteristics of spring and pond water from the study sites, February, 2015 – January, 2016

Ponds and springs	pH	Temperature (°C)	DO (mg/L)	CD (μS/cm)	Turbidity (TNU)
**WHO Acceptable levels**	**6.5–8.5**	**–**	**> 5**	**–**	**0–5**
*Dadira Pond*	7.34 ± 0.17	27.82 ± 0.43	7.72 ± 2.10	583.09 ± 75.11	46.67 ± 28.52
*Kibenge Pond*	6.87 ± 0.09	40.86 ± 0.47	6.28 ± 0.18	3280.83 ± 87.20	16.05 ± 2.56
*Mughende Pond*	8.46 ± 0.11	30.98 ± 0.44	12.2 ± 1.50	744.43 ± 32.15	102.74 ± 32.33
*Panyimur Pond*	7.21 ± 0.13	29.30 ± 0.56	3.98 ± 0.39	721.03 ± 154.56	22.06 ± 8.15
*Wanseko Pond*	5.73 ± 0.17	28.28 ± 0.88	4.10 ± 0.23	55.99 ± 7.75	116.16 ± 56.56
*Katanga spring*	6.19 ± 0.15	24.87 ± 0.36	4.65 ± 0.48	276.46 ± 45.46	41.71 ± 8.84
*Kibenge spring*	7.19 ± 0.06	41.89 ± 0.61	6.15 ± 0.17	3276.36 ± 81.88	5.68 ± 1.95
*Nyakirango spring*	7.27 ± 0.07	25.42 ± 0.99	6.80 ± 0.18	89.81 ± 16.63	120.75 ± 51.33

***NB: the values in the table are mean ± standard error.***

*DO* Dissolve Oxygen, *CD* Conductivity, *FLS* Fish Landing Site

The mean physicochemical characteristics of water from the springs and ponds showed variations between the sites. The majority of site means values were within the WHO accepted pH range. Two sites, Wanseko pond (Buliisa, district, Lake Albert basin) and Katanga spring (Kampala district, Lake Victoria basin) had mean water pH below the recommended WHO drinking water acceptable range at the acidic level of 5.73 and 6.19 respectively. Forty percent (40%, 2/5) of the ponds and 33% (1/3) of the springs had mean dissolved oxygen below the recommended WHO level. The ponds with the low dissolved oxygen were found within Lake Albert basin. Among the springs, Katanga spring (Kampala district, *L. victoria* basin) had mean dissolved oxygen that was below the WHO recommended level of 5 mg/L. Conductivities of the spring water were 89.81–3276.36 μS/cm and for ponds 55.99–3280.83 μS/cm. For both the springs and the ponds the differences between the lowest and the highest conductivities were wide.

### Monthly variations of the springs and ponds water physicochemical characteristics

**Fig. 4:**
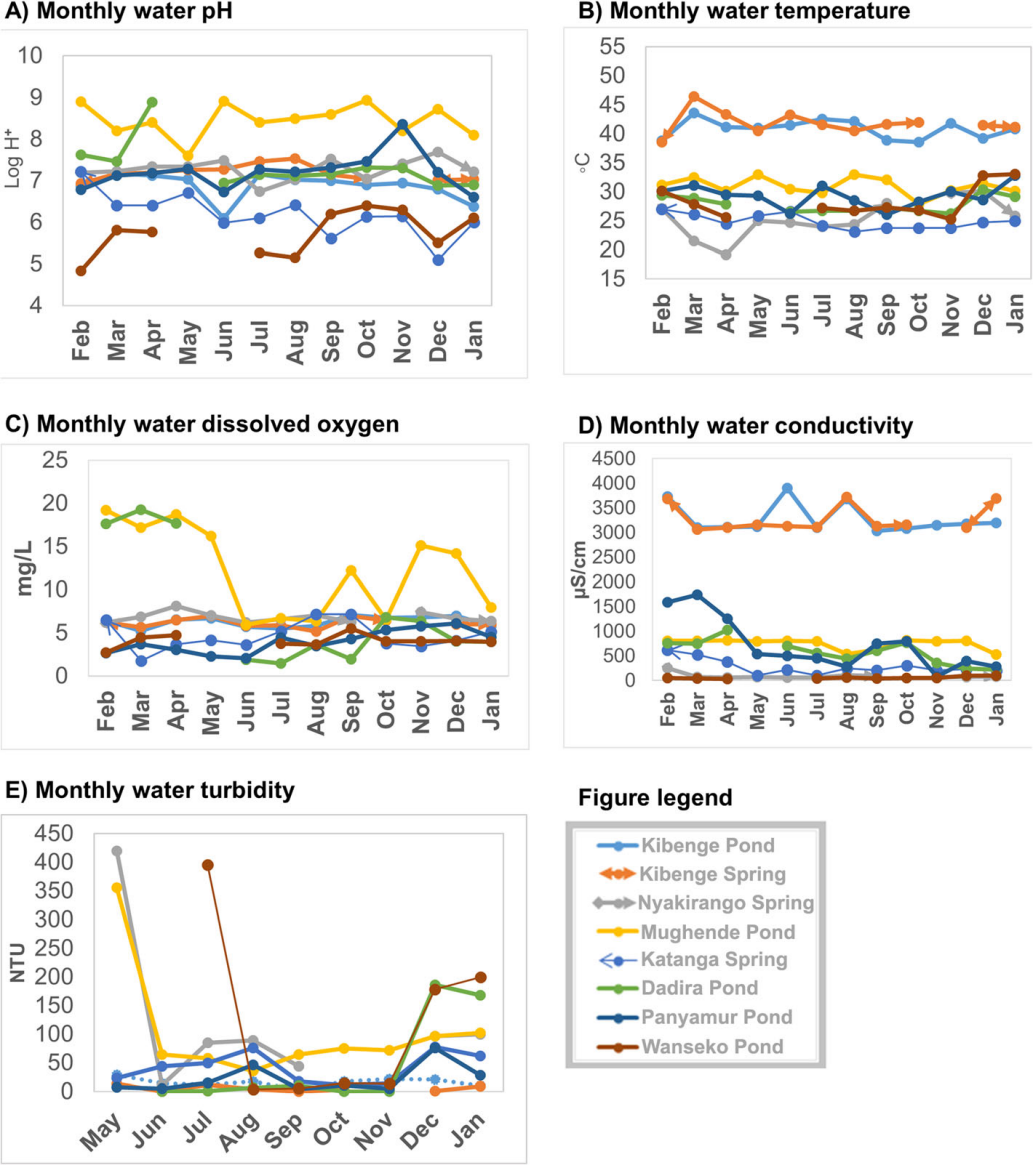
Monthly variations of the physicochemical characteristics of the spring and pond water, February 2015 – January 2016: Part **a**) water pH variations; Part **b**) water temperature variations; Part **c**) dissolved oxygen variations; Part **d**) conductivity variations and Part **e**) water turbidity variations

The monthly variations of spring and pond water physicochemical characteristics are shown in Fig. 4**.**

There were variations in the water physicochemical characteristics of the spring and the pond water overtime. The variations in water (springs and ponds) were also present between the different sites. The springs had small monthly variations of the water physicochemical parameters while the ponds had wide variations. Mughende pond (Kasese district) had the highest pH for most of the study period. Katanga spring (Kampala district) had the lowest pH compared to other springs during the study period. Kibenge spring (Kasese district) had higher temperature than the rest of the two springs (Katanga spring, Kampala district and Nyakirango spring, Kasese district). Most springs and ponds had slight fluctuations in dissolved oxygen except for Mughende pond (Kasese district). Most springs and ponds except for Panyimur pond (Nebbi district) had small monthly fluctuations in water conductivity. Kibenge spring and pond (both located in Kasese district) had higher conductivity compared to the rest of the springs or ponds. Mughende spring and pond were outliers with higher conductivity than the rest of the water sites. There were variations in water turbidity with months for both the springs and the ponds. Apart from Mughende pond (Kasese district), the rest of the springs and ponds showed variations that had two peaks, the first peak (May – August, 2015) and the second peak (November – January, 2016).

### Test results of the other surface water sources: Mobuku irrigation canal water

Mobuku irrigation canal water, water diverted from Mobuku River for irrigation purposes by the Mobuku irrigation scheme was tested because the local communities were using this water for domestic purposes including drinking. Apart from water turbidity which was above the WHO recommended standard of 5NTU, the rest of the water physicochemical parameters (pH, temperature, dissolved oxygen and conductivity) were in the WHO acceptable range as follow: pH of 7.93 ± Standard Error (SE) of 0.23, temperature of 26.57 °C ± SE of 1.25 °C, dissolved oxygen of 6.38 mg/L ± SE of 0.18 mg/L, conductivity of 69.06 ± SE of 2.57) and turbidity of 28.68 ± SE of 9.06NTU.

### Monthly variations of physicochemical characteristics of Mobuku irrigation canal water

**Fig. 5:**
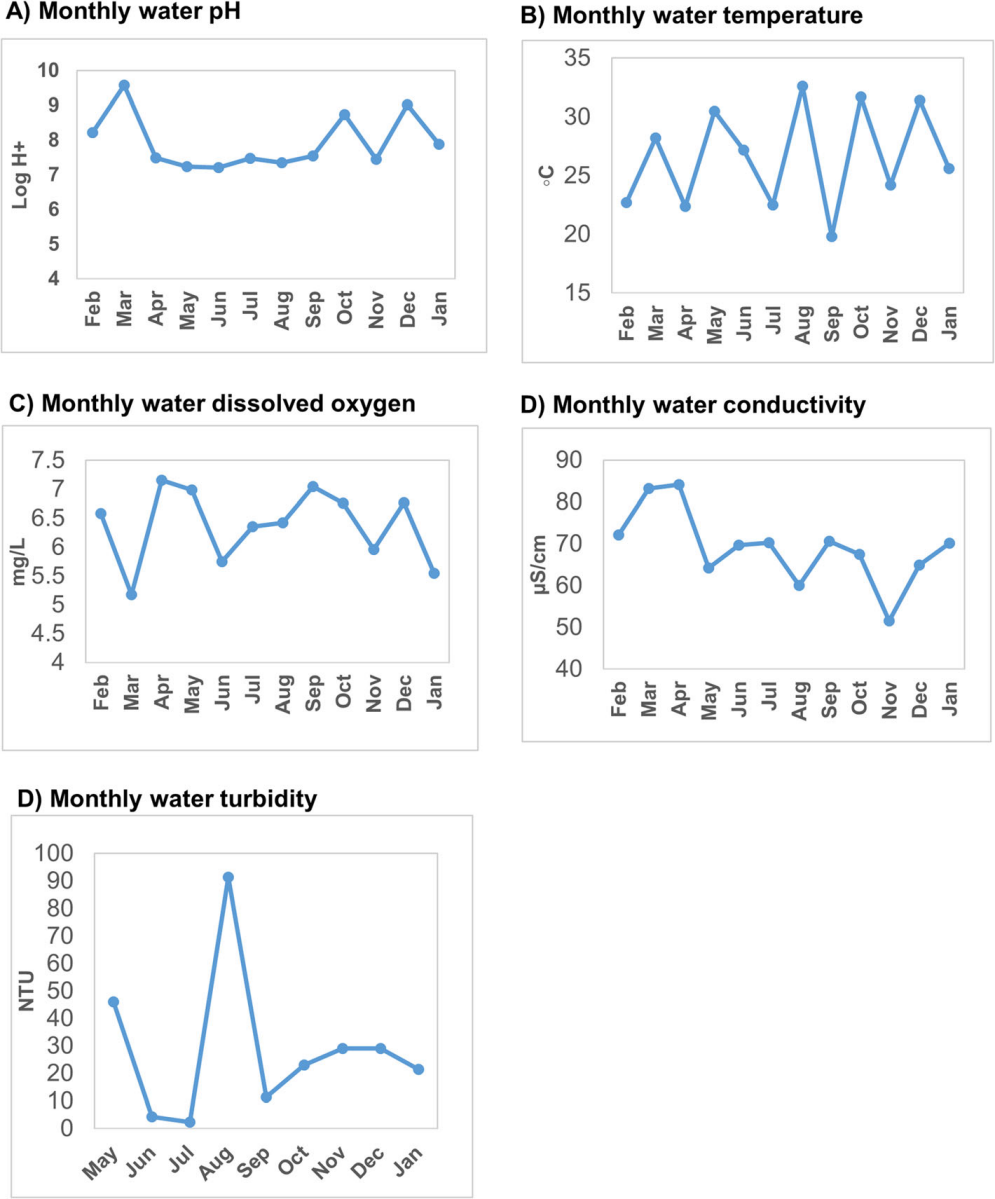
Monthly variations of the physicochemical characteristics of Mobuku irrigation canal water, February 2015 – January 2016: Part **a**) water pH variations; Part **b**) water temperature variations; Part **c**) dissolved oxygen variations; Part **d**) conductivity variations and Part **e**) water turbidity variations

There were monthly variations in water physicochemical characteristics of Mobuku irrigation canal. The water pH and dissolved oxygen showed two peaks each. The first peak was in March – May, 2015 and the second peak, August – November, 2015. The variations of the Mobuku irrigation canal monthly water physicochemical parameters over the study period is shown in Fig. 5**.**

### Results of statistical tests for the differences within sites overtime and between sites

*One-Way ANOVA test.*

There were no statistically significant differences within most of the study sites except for sites on the lakes and the rivers where the pH and temperature differences were statistically significantly within sites overtime. Statistically significant differences in the water physicochemical characteristics were observed between sites (all *p*-value < 0.05) as indicated in the additional file 2**.**

### Turkey’s post hoc test

There were statistically significant differences for all water physicochemical parameters for both the lake and river sites. For instance, Lake Edward had both the highest temperature (34 °C, May, 2015) which was registered at Katwe FLS (Kasese district) and the lowest temperature (18.9 °C, April, 2015) which was recorded at Kayanzi FLS (Kasese district). The results of the comparison of the physicochemical parameters of the various lake and river sites are shown in Table 4**.**

**Table 4:** Turkey’s post hoc test results for the comparison of the water physicochemical characteristics between sites on the lakes and the rivers in the study area

*Pairs for comparison*	*Widest or least difference*	*Difference*	*Standard error*	*t-statistic*	*P-values*
***pH***					
*Kahendero FLS (L. George)* vs *Port-Bell FLS (L. Victoria)*	Widest	−2.534	0.343	−7.39	0.000
*Panyimur FLS (L. Albert)* vs *Katwe Salt (L. Edward)*	Least	−1.113	0.335	−3.32	0.045
***Temperature***					
*Katwe Salt FLS (L. Edward)* vs *Kayanzi FLS (L. Edward)*	Widest	−7.500	0.982	−7.64	0.000
*Katwe Salt FLS (L. Edward)* vs *Kitwe FLS (L. Kyoga)*	Least	−3.250	0.982	−3.31	0.046
*R. Mobuku* vs *R. Victoria Nile*	Widest	6.125	1.318	4.65	0.000
*R. Mobuku* vs *R. Lhubiriha*	Least	4.133	1.318	−3.14	0.038
***Dissolved oxygen***					
*Port-Bell FLS* vs *Hamukungu*	Widest	−2.031	0.570	−3.57	0.021
*Port-Bell FLS* vs *Majanji*	Least	−1.981	0.570	−3.48	0.028
*R. Lubigi* vs *R. Lhubiriha*	Widest	−4.408	0.480	−9.18	0.000
*R. Victoria Nile* vs *R. Lhubiriha*	Least	−1.702	0.480	−3.54	0.012
***Conductivity***					
*Panyimur FLS (L. Albert)* vs *Kayanzi*	Widest	668.508	117.341	5.70	0.000
*Kalolo FLS (L. Albert)* vs *Hamukungu (L. George)*	Least	393.258	117.341	3.35	0.041
*R. Nyamugasani* vs *R. Mobuku*	Widest	882.933	37.880	23.31	0.000
*R. Victoria Nile* vs *R. Lubigi*	Least	− 319.684	37.880	−8.44	0.000
***Turbidity***					
*Kahendero FLS (L. George)* vs *Majanji FLS (L. Victoria)*	Widest	169.024	33.684	−5.02	0.000
*Kahendero FLS (L. George)* vs *Port-Bell FLS (L. Victoria)*	Least	112.302	33.684	−3.33	0.047
*R. Sio* vs *R. Mobuku*	Widest	128.593	29.500	4.36	0.001
*R. Nyamwamba* vs *R. Lubigi*	Least	−98.032	29.500	−3.32	0.025

Similarly, comparison of the springs or pond water showed statistically significant differences for most (80% of the total comparison) of the water parameters (pH, temperature, dissolved oxygen and conductivity) apart from the water turbidity. Turkey’s post Hoc test results for the comparison of springs and pond water physicochemical parameters are shown in Table 5.

**Table 5:** Turkey’s post hoc test results for comparison of the water physicochemical characteristics (pH, temperature, dissolved oxygen, conductivity and turbidity) for the springs and ponds that were studied

*Pairs for comparison*	*Widest or least difference*	*Difference*	*Standard error*	*t-statistic*	*P-values*
***pH***					
*Nyakirango spring* vs *Katanga spring*	Widest	1.084	0.147	7.360	0.000
*Nyakirango spring* vs *Kibenge spring*	Least	0.083	0.151	0.550	0.850
*Mughende Pond* vs *Wanseko Pond*	Widest	2.724	0.195	−13.960	0.000
*Mughende Pond* vs *Dadira Pond*	Least	1.118	0.190	5.870	0.000
***Temperature***					
*Katanga spring* vs *Kibenge spring*	Widest	17.018	0.968	17.580	0.000
*Katanga spring* vs *Nyakirango spring*	Least	0.545	0.968	0.560	0.841
*Kibenge* vs *Dadira pond*	Widest	13.040	0.791	16.480	0.000
*Wanseko* vs *Mughende pond*	Least	−2.703	0.812	−3.330	0.013
***Dissolved oxygen***					
*Katanga spring* vs *Nyakirango spring*	Widest	2.159	0.452	4.770	0.000
*Nyakirango spring* vs *Kibenge spring*	Least	0.657	0.462	1.420	0.342
*Panyimur* vs *Mughende pond*	Widest	−8.216	1.612	−5.100	0.000
*Mughende* vs *Kibenge pond*	Least	5.917	1.612	3.670	0.005
***Conductivity***					
*Nyakirango spring* vs *Kibenge spring*	Widest	− 3186.560	78.124	−40.790	0.000
*Nyakirango spring* vs *Katanga spring*	Least	−186.649	76.479	−2.440	0.052
*Wanseko* vs *Kibenge pond*	Widest	− 3224.843	131.695	−24.490	0.000
*Wanseko* vs *Dadira pond*	Least	− 527.101	134.388	−3.920	0.002
***Turbidity***					
*Nyakirango spring* vs *Kibenge spring*	Widest	115.071	38.545	2.990	0.019
*Kibenge spring* vs *Katanga spring*	Least	−36.026	36.189	−1.000	0.588
*Wanseko* Vs *Kibenge pond*	Widest	100.114	42.282	2.370	0.147
	Least	6.012	39.551	0.150	1.000

## Discussion

This study showed that water for drinking and domestic purposes from the surface water sources and springs in cholera affected communities/districts of Uganda were not safe for human use in natural form. The water samples from the water sources in the study area did not meet the WHO drinking water quality standards in terms of the important physicochemical parameters. In addition, all the surface water sources and the springs tested had turbidity above the WHO recommended level of 5NTU yet the same water were used for domestic purposes including drinking in the natural form by the households. The study also found variations in the other physicochemical parameters (pH, temperature, dissolved oxygen and conductivity) between study sites on the same lake and between the different water sources.

While the majority of the water sources had mean water physicochemical characteristics (excluding turbidity) in acceptable range, few water sources, mainly the sites on Lake George, including the springs and ponds had pH and dissolved oxygen outside the recommended WHO ranges. These water sources that did not meet the WHO drinking water standards could expose the users to harmful effects of unsafe drinking water including waterborne diseases such as cholera. The present study findings of high water turbidity if due to algae bloom could encourage pathogen persistence and infection spread, including *V. cholerae* bacteria [40, 41] resulting in ill-health and cholera epidemics. In addition, the high water turbidity complicates water disinfection as it gives rise to significant chlorine demand [53]. The increased chlorine demand can be costly and difficult to ensure constant availability for disinfection of water since Uganda and several other developing countries need and receive supplementary donor support [69].

In regard to temperature, dissolved oxygen and conductivity, the majority of the surface water sources and springs tested met the recommended WHO drinking water standards. However, a few water sources such as River Lubigi in Kampala district had mean dissolved oxygen below the recommended WHO drinking water standards. Therefore, in order to ensure universal access to safe drinking water, the water sources that had vital physicochemical parameters outside the WHO drinking water range could be targeted for further studies.

There were statistically significant differences in the water physicochemical characteristics between the different sites and sources (lakes, rivers, springs and ponds). Despite these differences, the required approaches to ensure safe water access to the communities may not differ across sites. First and foremost, all sites and water types will need measures that reduce the high water turbidity to WHO acceptable levels. Secondly, in few instances, such as the water sources with pH in acidic range (Katanga spring in Kampala district, Lake Victoria Basin and Wanseko pond in Buliisa district, lake Albert basin) in addition to requiring further studies to identify the causes of the low pH (acidity), such water sources may also require the use of water treatment methods that neutralize the excess acidity [54]. Furthermore, since acidity is usually associated with increased solubility of toxic heavy metals (lead, arsenic and others) [34], testing such water for metallic contamination may be required. Heavy metal contamination of water causes ill-health due to chronic exposure which is cumulative and manifest late for correction to be done [70].

The findings of this study also highlight the differences in water quality between the urban surface water sources and springs (Kampala district) and the rural surface sources and springs (other study districts – Kasese, Kayunga, Busia, Nebbi and Buliisa) The water sources that met the WHO recommended drinking water quality standards [53] were mostly the rural springs and the rivers. However, these differences between the rural and the urban water sources do not alter the required approaches to ensure access to safe water which is by promoting measures that reduce the high water turbidity in combination with water disinfection to remove the pathogens. The relatively good quality of rural water sources compared to the urban ones could have been due to availability of plenty of vegetation in rural setting that filtered the water along the way downstream and possibly low level of pollution from industrial inputs in rural areas than in urban areas [71, 72].

In relation to cholera outbreaks in the study communities, naturally, the physicochemical conditions for survival of *V. cholerae O1* occur in an estuarine environment and other brackish waters [73, 74]. In such circumstances, the favourable physicochemical conditions for *V. cholerae* isolation are the high water turbidity [49] and temperature of above 17 °C [43]. Interestingly, all the surface water sources and the springs tested had favourable physicochemical characteristics for the survival of *V. cholerae* in terms of these two parameters (high water turbidity and temperature of above 17 °C). Furthermore, two lakes sites (Kahendero FLS and Hamukungu FLS, Lake George, Kasese district) had also favourable mean pH for the survival of *V. cholerae* of 9.03 ± 0.17 and 9.13 ± 0.23 respectively. Favourable pH for *V. cholerae* survival in waters of Lake George was previously documented in the same area [23]. Hence, the frequent cholera outbreaks [19–21, 24] in the study area could be attributed to both the favourable physicochemical water characteristics and use of unsafe water.

There were wide variations in conductivity between water sources and within the same source overtime. High water conductivities were recorded in the months of January to March 2015 (dry season), possibly due to high evaporation which increased the concentration of electrolytes present in water. Likewise, two rivers namely. River Lubigi (Kampala district) and Nyamugasani (Kasese district) had higher mean conductivities of 460.51 ± 57.83 μS/cm and 946.08 ± 3.63 μS/cm respectively than for typically unpolluted river of 350 μS/cm [75]. Consequently, given that the two rivers flow through areas of heavy metal mining (copper and cobalt mines in Kasese district by Kilembe Mines Limited and Kasese Cobalt Company Limited) and industrial activities (Kampala City), it is possible for the high water conductivity to be due to the heavy metal contamination as previously documented in drinking water in South-western Uganda [62] and Kampala City [61]. Thus, specific studies are required on water from the two rivers to determine the true cause of the high conductivity and to guide mitigation measures.

Hence, more efforts are required to promote safe water access in Uganda to attain the WHO cholera elimination target [25] and SDG 6 by 2030 since 26% (36/135) of mean physicochemical water tests did not meet WHO drinking water quality standards [53]. These findings together with those of the previous studies which demonstrated the presence of pathogenic *V. cholerae* in the same water sources [22, 23, 76] should guide stakeholders to improve access to safe water in the Great Lakes basins of Uganda holistically. Thus, measures such as promotion of use of safe water (using water disinfection), health education, sanitation improvement and hygiene promotion that address both the water bacteriological contents and physicochemical parameters should be considered in both the short and medium terms. However, long term plan to increase access to safe water by construction of permanent safe water treatment plants and distribution systems (pipes) should remain a top priority.

In the short and intermediate period, focusing on the measures that reduce water turbidity and disinfection of water (to kill microorganisms) should be prioritized so as to facilitate progress towards attainment of SDGs and cholera elimination in the study area. The basis for such prioritization lies in the fact that high water turbidity raises water temperature and prevents the disinfection effects of chlorine on water. These in return promote survival of the microorganisms and consequently cholera and other waterborne disease outbreaks. Furthermore, though boiling of water is feasible and recommended through technical guidelines [26] since it addresses both turbidity and kills the micro-organisms, it has issues of poor compliance due to lack of firewood which is the main cooking energy source in these communities [70]. Therefore, alternative safe water provision targeting reduction of high water turbidity and removal of microorganism by special filters such as decanting and sand filters and flocculation agents which do not need heat energy should be promoted [77, 78]. Also, there is a need to explore the use of solar energy (solar water purifiers) [79] in these communities given their location in the tropics where sunshine is plenty. In the minority of situations, in addition to use of above methods to make water safe, there may be a need to employ different approaches of water purification depending on the water source. For example the water sources with lower or higher than recommended pH [53] (Wanseko pond, Hamukungu and Kahendero FLS on L. George), use of water treatment reagents that are affected by pH such as chlorine tablets should be reevaluated.

In additional to disinfection and turbidity corrective measures for all the water that were studied, each of the springs in the study area (Katanga in Kampala district and Nyakirango and Kibenge springs in Kasese district) will also need a sanitary survey (a comprehensive inspection of the entire water delivery system from the source to the mouth so as to identify potential problems and changes in the quality of drinking water) [80]. The findings of the sanitary survey should then guide the medium and long term interventions for water quality improvement in areas served by targeted springs. The following are some of the interventions that could be carried out after a sanitary survey: provision of a screen to prevent the entrance of animals, erecting a warning signs, digging of a diversion ditch located at the uphill end to keep rainwater from flowing over the spring area, establishment of an impervious barrier (a clay or a plastic liner) to prevent potential contaminants from entering into the water or and others measures described in the handbook for spring protection [81].

Furthermore, as a stopgap measure while access to safe water is scaled up, the communities in the study area should be protected from cholera using Oral Cholera Vaccines [82]. Protection of these communities is necessary since this study shows that favorable conditions for cholera propagation/transmission are present in the water in the study area. The favorable conditions that were documented in this study included the high water turbidity which makes it difficult to disinfect water [53] and the water temperature of above 17 °C which speeds up the multiplication of pathogens [43].

In addition, there were some other important study findings that were not fully understood. For example, some water sources (Kibenge spring and pond (located in Kasese district, western Uganda) had extreme vital physicochemical values for both conductivity and water temperature relative to the rest of above 40 °C and 3000 μS/cm respectively. It is possible that the extreme values were due to geochemical effects documented in water sources around Mount Rwenzori [83]. However, since there was copper and cobalt mining in Kasese district, high water conductivity could have been due to chemical contamination. Similarly, River Lubigi, Kampala district (central Uganda) had very low dissolved oxygen of less than 1 mg/L during some months (for example in January 2015, dissolved oxygen of 0.45 mg/L) which could have been due to organic pollutants from the communities in Kampala City [84] that used up the oxygen in the water. Also, Wanseko pond (Lake Albert basin, Buliisa district) had low pH of 4.84 in February 2015. Such water with low pH have the potential to increase the solubility of heavy metals some of which make water harmful when consumed [85]. Therefore, further studies will be required to better understand such extreme values.

### Strength and limitations of this study

This study had several strengths. First, the longitudinal study design that employed repeated measurements of water physicochemical characteristics from the same site and source. This design reduced the likelihood of errors that could arise from one-off measurements seen in cross-sectional study designs resulting in increased validity of the study findings. Second, the inclusion of a variety of the water sources from which drinking and domestic water were collected namely, lakes, rivers, ponds, springs and a canal from different regions of Uganda made the findings representative of the water sources in study districts. Third, use of robust equipment, *Hach meters, HQ40d* [68] which automatically compensated for the weather changes (corrected for possible confounders and biases) for the parameters that had effect on each other such as raising water temperature impacting on the water conductivity and dissolved oxygen. Forth, purposive selection of the districts with frequent cholera outbreaks, an important waterborne disease that is targeted for elimination locally within Uganda and globally by WHO [25]. This meant that the findings had higher potential for used by stakeholders targeting to improve access to safe water and those for cholera prevention.

There were also some study limitations. First, though the study identified the favourable conditions (higher than recommended mean water turbidity and temperature of above 17 °C) for cholera in the study area, we could not report on causal-effect relationship between *V. cholerae* and the parameters studied. *Vibrio cholera*e pathogens were detected by use of multiplex Polymerase Chain Reaction (PCR). The results for PCR test were interpreted as positive or negative for *V. cholerae O1, O139, non O1, and non O139* [22]. These data were not appropriate for establishment of causal-effect relationship Therefore, further studies using appropriate methods are recommended to establish such relationships.

Second, during some months of the study, water samples could not be obtained from some sources especially the ponds that had dried up during the dry season. The drying up reduced the number of samples collected from these points. However, since the months without water were few compared to the entire study period, the impact of the missing data could have been minimal.

Third, water samples were only tested for the five key physicochemical water characteristics, Vital Signs [32] however, there are many other parameters that effect survival and health of living things namely, nitrates, copper, lead, fluoride, phosphates, arsenic and others. Studies are therefore required to provide more information on these other parameters not addressed by the current study.

## Conclusions

The study showed that surface and spring water for drinking and other domestic purposes in cholera prone communities in Great Lakes basins of Uganda were unsafe in terms of vital physicochemical water characteristics. These water sources had favourable physicochemical characteristics for transmission/propagation of waterborne diseases, including cholera. All test sites (100%, 27/27) had temperature above 17 °C that is suitable for *V. cholerae* survival and transmission and higher than the WHO recommended mean water turbidity of 5NTU. In addition, more than a quarter (27%) of lake sites and 40% of the ponds had pH and dissolved oxygen outside the WHO recommended range of 6.5–8.5 and less than 5 mg/L respectively. These findings complement bacteriological findings that were previously reported in the study area which found that use of this water increased their vulnerability to cholera outbreaks [22]. Therefore, in order for Uganda to attain the WHO cholera elimination and the United Nations SDG 6 target by 2030, stakeholders (the Ministry of Water and Environment, the local governments, Ministry of Health development partners and others) should embrace interventions that holistically improve water quality through addressing both physicochemical and biological characteristics. Furthermore, studies should be conducted to generate more information on the other physicochemical parameters not included in this study such as detection of the heavy metal contamination.

## Supplementary information

**Additional file 1.** The number and the type of water sources in each of the lake basins in cholera prone communities of Uganda that were enrolled in the study, February 2015 – January 2016.

**Additional file 2.** One Way ANOVA test results for the differences within the study sites overtime (February 2015 – January 2016) and between sites.

## Data Availability

The datasets generated and/or analysed during the current study are available in the Mendeley Data repository, 10.17632/57sw2w23tw.1. The cholera incidence data used to identify the study area were from Uganda Ministry of Health and the district (Kasese, Busia, Nebbi, Buliisa and Kayunga) weekly epidemiological reports.

## Abbreviations

ANOVA: Analysis of Variance
CD: Conductivity
CPHL: Central Public Health Laboratories
DO: Dissolved Oxygen
DOVE: Delivery of Oral Vaccines Effectively
FLS: Fish landing site
IRB: Institutional Review Board
MOH: Ministry of Health
NTU: Nephrometric units
PCR: Polymerase Chain Reaction
SDG: Sustainable Development Goal
SOPs: Standard Operating Procedures
Turb: Turbidity
USA: United States of America
WHO: World Health Organization
