# Supplementary material for: The quality of drinking and domestic water from the surface water sources (lakes, rivers, irrigation canals and ponds) and springs in cholera prone communities of Uganda: an analysis of vital physicochemical parameters
Source: BMC Public Health. 2020 Jul 17;20:1128. doi: 10.1186/s12889-020-09186-3 (PMC7368733; doi:10.1186/s12889-020-09186-3)
Supplement: Supplementary file 1 — Additional file 1. The number and the type of water sources in each of the lake basins in cholera prone communities of Uganda that were enrolled in the study, February 2015 – January 2016. [file 12889_2020_9186_MOESM1_ESM.doc]

Additional file 1. The number and the type of water sources in each of the lake basins in cholera prone communities of Uganda that were enrolled in the study, February 2015 – January 2016.

| **Lake** | **Number of test sites** | | | | **Study site** |
| --- | --- | --- | --- | --- | --- |
| **Lake basin** | **Lake shore sites** | **River sites** | **Springs and ponds** | **Other water types (canal)** | **District** |
| **Lake Victoria** | 3 | 2 | 2 | 0 | Kampala and Busia |
| **Lake Kyoga** | 2 | 0 | 0 | 0 | Kayunga |
| **Lake Albert** | 2 | 1 | 2 | 0 | Nebbi and Buliisa |
| **Lake Edward** | 2 | 2 | 2 | 0 | Kasese |
| **Lake George** | 2 | 2 | 2 | 1 | Kasese |
| **Total** | **11** | **7** | **8** | **1** |  |
