# Supplementary material for: The quality of drinking and domestic water from the surface water sources (lakes, rivers, irrigation canals and ponds) and springs in cholera prone communities of Uganda: an analysis of vital physicochemical parameters
Source: BMC Public Health. 2020 Jul 17;20:1128. doi: 10.1186/s12889-020-09186-3 (PMC7368733; doi:10.1186/s12889-020-09186-3)
Supplement: Supplementary file 2 — Additional file 2. One Way ANOVA test results for the differences within the study sites overtime (February 2015 – January 2016) and between sites. [file 12889_2020_9186_MOESM2_ESM.doc]

**Additional file 2. Table of the results of One Way ANOVA test for the difference within the sites over**time (February 2015 – January 2016) and between sites

|  | **Difference over time** | | **Difference between sites** | |
| --- | --- | --- | --- | --- |
| ***Site*** | **F** | **P-value** | **F** | **P-value** |
| ***Lake sites*** |  |  |  |  |
| *pH* | (11, 108) = 6.5 | 0.000 | (10, 108) = 20.01 | 0.000 |
| *Temperature* | (11, 108) = 4.14 | 0.001 | (10, 108) = 10.19 | 0.000 |
| *Dissolved oxygen* | (11, 109) = 0.69 | 0.701 | (10, 109) = 3.09 | 0.002 |
| *Conductivity* | (11, 108) = 1.27 | 0.252 | (10, 108) = 8.85 | 0.000 |
| *Turbidity* | (8, 77) = 0.79 | 0.613 | (10, 77) = 4.06 | 0.000 |
| ***River sites*** |  |  |  |  |
| *pH* | (11, 83) = 2.81 | 0.036 | (6, 83) = 3.27 | 0.007 |
| *Temperature* | (11, 108) = 4.14 | 0.001 | (6, 82) = 6.2 | 0.000 |
| *Dissolved oxygen* | (11, 83) = 0.98 | 0.474 | (6, 83) = 19.80 | 0.000 |
| *Conductivity* | (11, 83) = 1.49 | 0.156 | (6, 83) = 159.26 | 0.000 |
| *Turbidity* | (8, 48) = 0.55 | 0.814 | (6, 48) = 5.82 | 0.000 |
| ***Springs*** |  |  |  |  |
| *pH* | (11, 21) = 0.47 | 0.899 | (2, 21) = 27.17 | 0.000 |
| *Temperature* | (11, 20) = 0.44 | 0.921 | (2, 20) = 152.99 | 0.000 |
| *Dissolved oxygen* | (11, 20) = 0.70 | 0.726 | (2, 20) = 10.36 | 0.001 |
| *Conductivity* | (11, 20) = 1.77 | 0.129 | (2, 20) = 1296.92 | 0.000 |
| *Turbidity* | (8, 13) = 0.77 | 0.636 | (2, 13) = 3.81 | 0.050 |
| ***Ponds*** |  |  |  |  |
| *pH* | (11, 41) = 1.27 | 0.278 | (4, 41) = 53.95 | 0.000 |
| *Temperature* | (11, 41) = 1.93 | 0.063 | (4, 41) = 112.01 | 0.000 |
| *Dissolved oxygen* | (11, 41) = 1.95 | 0.060 | (4, 41) = 10.49 | 0.000 |
| *Conductivity* | (11,41) = 1.70 | 0.107 | (4, 41) = 222.20 | 0.000 |
| *Turbidity* | (8, 29) = 1.70 | 0.141 | (4, 29) = 3.02 | 0.034 |
